# Physical Activity, Screen Time, Sedentary and Sleeping Habits of Polish Preschoolers during the COVID-19 Pandemic and WHO’s Recommendations: An Observational Cohort Study

**DOI:** 10.3390/ijerph182111173

**Published:** 2021-10-24

**Authors:** Anna Brzęk, Markus Strauss, Fabian Sanchis-Gomar, Roman Leischik

**Affiliations:** 1 Department of Physiotherapy, Faculty of Health Sciences, Medical University of Silesia, 40-752 Katowice, Poland; 2 Department of Cardiology I—Coronary and Peripheral Vascular Disease, Heart Failure Medicine, University Hospital Muenster, Cardiol, 48149 Muenster, Germany; markus.strauss@ukmuenster.de; 3 Department of Cardiology, Faculty of Health, School of Medicine, University Witten/Herdecke, 58095 Hagen, Germany; roman.leischik@uni-wh.de; 4 Division of Cardiovascular Medicine, School of Medicine, Stanford University, Stanford, CA 94305, USA; fabian.sanchis@uv.es

**Keywords:** children under 5, sedentary lifestyle, COVID-19 pandemic

## Abstract

Background: Restrictions related to the COVID-19 pandemic may lead to a significant decrease in physical activity, an increase in sedentary behavior, and thus also such things as screen time or a change in health behavior patterns. The survey aimed to compare levels of physical activity, screen time, hours spent sitting and sleeping time among Polish children aged 3–5 years of age before and during the COVID-19 pandemic. Methods: We identified 3000 respondents under five years of age, at Polish kindergartens. The questionnaire consists of 62 questions according to the recommendations of health behavior in school-aged children. The questionnaire was completed by the parents of these children. Results: Only 30.77% of children complied with WHO criteria before the pandemic. During the pandemic, the percentage of children meeting the recommendations for physical activity decreased even more. Children spent much more time in a sitting position before the restrictions. The children slept as recommended 10–13 h a day, and the pandemic caused an increase in sleep duration of 10–18%. Most children had a limited time allowed for the use of electronic devices already before the pandemic, but during the pandemic the results negatively decreased by 71.54%. Conclusions: The results clearly indicate decreased physical activity and increased screen time. It is also crucial to develop recommendations for prevention management strategies of sedentary lifestyles in the youngest group.

## 1. Introduction

Following less than two years of the COVID-19 pandemic with varying degrees of severity, health consequences can be identified. Restrictions and quarantine related to the pandemic may lead to a significant increase in sedentary behavior [1,2]. The consequence may be increased use of electronic devices or modified health behavior patterns, such as sleep disturbances or decreased physical activity (PA) [3,4]. The time spent sitting may be different on common days than on weekend days, which gives a wider picture of the sedentary positions taken during leisure time that could be used for different forms of PA.

For this age group, according to the new 2019 World Health Organization (WHO) recommendations, special attention should be paid to ensure that an appropriate dose of physical activity, sitting behavior, and electronic device use and sleeping time is maintained for proper psychomotor development [5,6]. According to these recommendations, a child needs at least 180 min of PA daily, including a minimum of 60 min of moderate to vigorous PA, a maximum of 60 min in a sitting position, as well as 60 min of screen time. Time allocated for sleep is in the range of 10–13 h of sleep per night [5,6]. The recommendations are based on the need for movement and due to the advantage of the sympathetic nervous system over the parasympathetic one (stimulation over inhibition) [7]. WHO recommendations before the COVID-19 pandemic have become a priority in educating the public about the health consequences of sedentary lifestyles [5,6]. Sifting through the literature indicated that this issue has not yet been well defined in a group of children, especially those of a preschool age. The papers found concern following a dynamic increase in children’s body weight, who see the main cause in the reduction of PA and changes in exercise habits in recent times, including the lockdown period [8,9,10]. Such a problem was pointed out by the Commission on Ending Childhood Obesity and they identified the need for guidance on PA, particularly for early childhood (<5 yo), a period of rapid physical and cognitive development [11]. Several publications were also found concerning mainly significant decreases in PA and increases in sedentary behavior. Social restrictions, including kindergarten education and ‘shelter-at-home’ recommendations, have made it difficult for children to engage in PA, sports, or other forms community-based organized PA [4,8,12]. Preliminary scientific evidence following the start of the COVID-19 pandemic-related restrictions showed a significant increase in screen time consumption [8,13,14]. Other papers have focused on sleep schedules/sleep quality in children as well as the suggested WHO recommendations [1,15,16]. They aimed to provide strategies for permanent behavior change in order to ensure the health and well-being of children during the COVID-19 pandemic, as well as indications of conditions enabling preschoolers to practice healthy movement behaviours and to meet the global guidelines [8,17,18,19,20]. Young children are directly influenced by their environmental patterns and family behavior. During the pandemic, it was up to them how to spend their free time, participation in sports, or health habits [4]. It is also important to remember that the development of new technologies caused their frequent use in leisure time [10,21]. Considering the aforementioned and the prolonged periods of social isolation associated with the COVID-19 pandemic and activity based on new technologies, there is a risk of perpetuating unhealthy lifestyles, leading to difficulties in readaptation after the COVID-19 crisis [22]. It is therefore important to assess the risk factors for the youngest group of children. 

There is still little of literature on lifestyle during a pandemic concerning preschoolers during a pandemic versus a prepandemic period [20,23]. The overall objective of the project is to recognize the scale of the problem in Poland in terms of the variety of patterns and health behavior of today’s children and possible changes during limitations caused by the COVID-19 pandemic. Appropriate research questions were formulated, as presented below:

1. How much time do children spend in a sitting position during the COVID-19 pandemic compared to the pre-pandemic period?
2. What is the level of PA in children during the pandemic? And has the volume of PA changed and what were the changes?
3. Is there a lockdown relationship with change in electronic device use habits compared to before the pandemic?
4. Have the behavior and health habits regarding sleeping changed during the pandemic in comparison with the period before it began? What have these changes been about?
5. Have WHO recommendations for hours of PA, sitting and sleeping been met?

## 2. Materials and Methods

### 2.1. Study Setting

**Figure 1:**
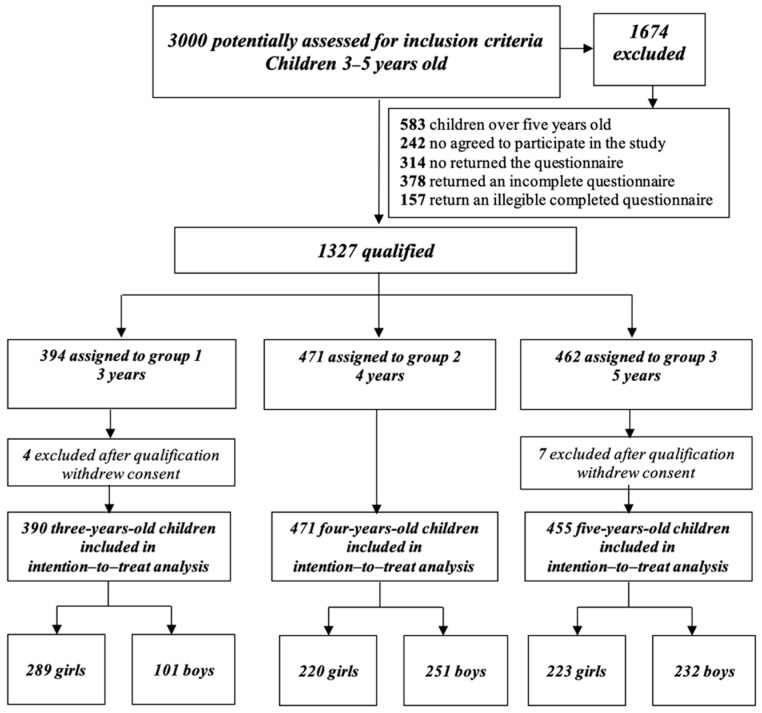
Flow diagram for selection and inclusion criteria.

The research was carried out with more than 3000 respondents under five years of age in Poland. Electronic and paper versions were available, depending on the form preferred by the children’s parents (Figure 1). Questionnaires correctly and fully completed by parents of children aged 3–5 years were qualified for final analysis. Participants were screened out if a child had been diagnosed with COVID-19 or if the family was in self-isolation or quarantine due to COVID-19. After consideration of inclusion and exclusion criteria, 1316 children were included in the final analysis (732 girls and 584 boys). The average age of parents completing the questionnaire was 34.62 ± 5.01.

### 2.2. Research Area—Obtaining the Respondents

The permissions were obtained in educational institutions in Silesia. Requests were sent to the heads of appropriate institutions—departments of education, departments of education in Silesian cities: Katowice, Mikołów, Mikołów district, and Chorzów. The cover letters to the directors of kindergartens were attached with a description of the project and questionnaires. Cover letters were sent by Electronic Platform for Public Administration Services (*pol*. ePUAP) or e-mails by the department to all educational institutions in the city that had agreed to conduct the research.

### 2.3. Questionnaire

The questionnaire consisted of 62 questions and was completed by parents. The questions concerning PA and sedentary behavior were arranged according to the recommendations of Health Behavior in School-aged Children (HBSC) [24,25]. The first part included questions on metrics on gender, age, body height, and weight. The following sections included questions to indicate normal, healthy behavior. The second part was concerned with PA and its changes during the restrictions related to the COVID-19 pandemic. The questions posed in the survey were very detailed and concerned the determination of the time (in minutes), frequency of attendance at organised sports activities before the pandemic (number of days per week) and forms of their replacement during the period of epidemic risk (by indicating specific forms of PA mentioned in the questionnaire, such as riding a bike, scooter, skateboard, playing ball, jumping rope, trampoline, dancing, or another activity with the possibility of a parent openly completing a specific PA. The third part related to sedentary time and its changes during the restrictions related to the COVID-19 pandemic. The questions asked in the survey concerned the determination of time, frequency of use of electronic devices, and their type. Questions were dedicated separately for the time before the pandemic and for the pandemic period. For each electronic device e.g., television (TV), personal computer (PC), tablet, computer, mobile, active games), the number of days of use and the time of use (in minutes) were indicated separately. The questions concerned the use of these devices during the week, from Monday to Friday and on weekends. Assuming a sitting position during the day (in minutes) separately for playing, learning, eating, resting for each day of the week separately. The questions about, for example, limiting time on electronic devices, holding correct positions while learning and playing, were determined on a six-point scale (5-always, 4-usually, 3-often, 2-sometimes, 1-rarely, 0-never). The fourth part included a few questions about sleeping behaviors during the pandemic period in comparison to the previous period. A question was asked about the number of hours of sleep per 24 h.

### 2.4. Data Collection—Stages

Data collection and recruitment for this study occurred from April–November 2020 in the Silesia District of Poland. Pandemic restrictions during this data collection period began with a ban on social gatherings outside of the household on March 2020, which remained in place until March of 2021 [26]. The typical school year in the region spans September–June. Because of the pandemic, in-person classes ceased on 11 March 2020, and the kindergartens opened for the new school year in early September 2020. Unfortunately, starting in September, kindergarten facilities were closed depending on the province and the rate of increasing COVID-19 infections. Most of the establishments in Silesia were closed during the study period.

Stage I assessment of the reliability of the measurement tool (double measurements of the same variable) using Cohen’s Kappa coefficient was based on 67 questionnaires completed by parents at intervals of several days at different times of the day to see if, for example, the fatigue factor affected the results. A test-re-test was used for this purpose. The first survey was conducted on 14–15 April 2020, and the second survey was conducted on 25–26 April 2020. Reproducibility of the test for each question was assessed by checking basic normality, and tests of homogeneity and sphericity were applied, as well as the Friedman test if the normal distribution criterion was not met. The significance of univariate ANOVA tests with Greenhouse-Geisser and Huynh-Feldt correction, if any, were evaluated sequentially (F = 0.089, *p* = 0.914 with GG correction = 0.9, *p* = 0.89).

Stage II consisted of conducting the main research between May–June 2020 and on 18 November 2020. Potential respondents were sent an email link to the survey or the link was posted on the preschool’s website, and the survey took about 15–20 min to complete. Parents with more than one child completed the questionnaire separately for each child. After data collection was complete, data were sifted from those surveys erroneously filled out and from incomplete questionnaires. The coding of descriptive data into numerical data began in the following sequence.

Stage III Preparation of Flow-diagram (cf. Figure 1) and making many analyses taking into account the divisions into groups in terms of:

- Gender: girls/boys
- Age: children 3–5 years old—pre-school group
- PA of groups: (A) active and (B) sedentary lifestyle The active group including guidelines on PA (MVPA—Moderate Vigor Physical Activity), were adapted according to WHO recommendations for children 3–4 years of age who (1) spend at least 180 min on a variety of types of physical activities at any intensity, of which at least 60 min is moderate- to vigorous-intensity PA, spread throughout the day; (2) are not restrained for more than 1 h at a time (e.g., prams/strollers) or sit for extended periods of time. Sedentary screen time should be no more than 1 h. The sedentary lifestyle group included children who did not meet the *MVPA* criteria
- BMI-for-age (Body Mass Index) status categories and the corresponding percentiles were based on WHO’s growth standard references. The cut-offs interpretations by the standard of the International Obesity Task Force (IOTF) were defined: (1) obesity with values > 2 SDs (Standard Deviations); (2) overweight > 1 SDs; (3) normal with values 1 ≥ z-score BMI ≥ −2; (4) thinness < −2SDs; (5) severe thinness < −3SDs [27,28].

### 2.5. Ethics Approval

The study was conducted according to the provisions of the Helsinki Convention, and the Bioethics Committee of the Medical University of Silesia Katowice expressed its approval (Decision No.: PCN/0022/KB/87/20; 15 May 2020).

### 2.6. Statistical Analysis

For single pieces of missing data, a method was used to supplement it with data from the first pre-pandemic investigation. Quantitative data were described using mean values and standard deviation. Homogeneity between samples were examined using the Kolmogorov–Smirnov 2-sample test and, if necessary, supplemented by the Lilliefors revision. Baseline characteristics of both groups were compared using 2-sample *t*-tests for continuous variables and the chi-square test for categorical variables. It was calculated at 95% confidence intervals (CIs). The following statistics methods were used for data analysis: Mann–Whitney *U* test for continuous variables with abnormal distribution, Student’s *t-*test for continuous variables with normal distribution—to assess relations between examination and non-parametric characteristics of the test χ^2^, and Spearman’s rank test and ANOVA tests and regression analysis.

Paired *t*-tests for complete cases assessed changes in sitting time for the week and the weekend, screen time (weekly frequency, total duration with leisure screen time), and sleep outcomes including daytime sleep time, total PA (weekly duration, weekly frequency, total duration, home-based duration). The total time of PA included outdoor play in the yard or street around the house (weekly duration); outdoor play in a park or playground or outdoor recreation area (weekly duration); and active indoor play at home (weekly duration).

All statistical analyses were performed using Statistica for Windows, version 13.3 (TIBCO Software Inc., Palo Alto, CA, USA) and the 2-tailed 5% level of significance were performed.

## 3. Results

### 3.1. Anthropometrics Parameters during Pandemic

A total of 3000 participants completed the questionnaire, and after validation of the data, 1316 respondents were included in the study, aged between three and five years (732 girls and 584 boys). The vast majority of the examined children lived in urban areas (83.89%) and rural areas (16.11%). The cut-offs interpretations were defined as, respectively: obesity with values >2 SDs was observed in 4.10%; overweight > 1 SDs was found in 8.81%; normal with value 1 ≥ z-score BMI ≥ −2 was noted at 65.58%; thinness < −2SDs and severe thinness <−3SDs observed in 21.42%. The z-score parameter did not change significantly during the pandemic (F = 2.018, *p* = 0.089). Participants’ general characteristics and anthropometrics are presented in Table 1.

### 3.2. Sedentary Habits Changes during COVID-19 Pandemic

Before the pandemic, children were in a sedentary position on average 2 h a day and about 3 h a day during the COVID-19 pandemic. The greatest differences were observed between the groups of three and four-year-olds and a group of five-year-olds (1/3 0.01; 2/3 0.001). Only slightly more than 20% of the children in each group met the WHO recommendations, which still decreased significantly during the pandemic (Table 2).

Regardless of the period, about 50% of children (3 yo: 47.43%; 4 yo: 46.17%, 5 yo: 56.48%) were in a sitting position in the cross-legged sit. About 30% used a functional ball for this position (3 yo: 31.88%; 4 yo: 27.23, 5 yo: 28.78%). Children rarely assume a bent position (3 yo: 68.72%; 4 yo: 62.34%, 5 yo: 61.97%). More than 60% of children’s parents paid attention to awkward sitting positions (3 yo: 61.02%; 4 yo: 63.83%, 5 yo: 65.93%).

### 3.3. Physical Activity Changes during COVID-19 Pandemic

**Figure 2:**
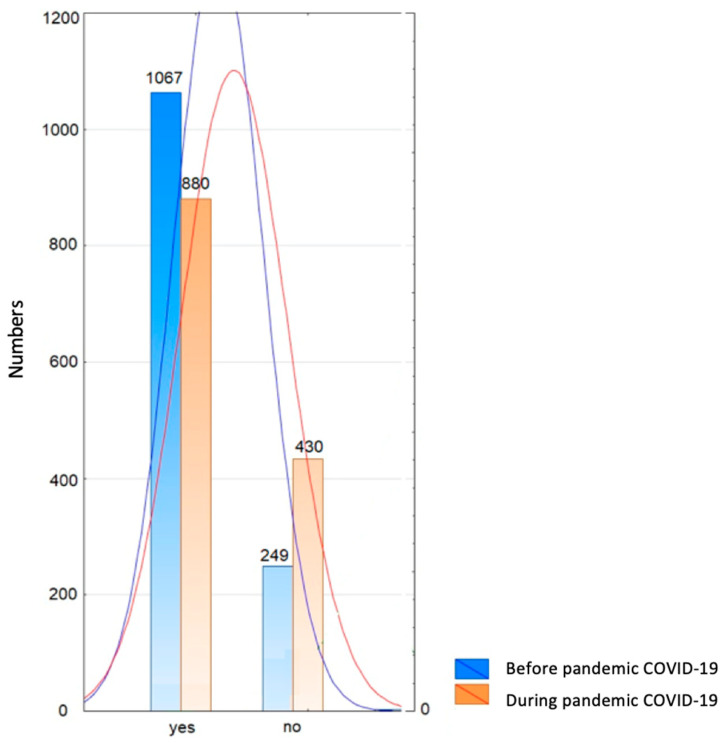
Number distribution of active and inactive children aged 3–5 years before and during the pandemic (yes—actively spent time; no—time not actively spent).

More than 34% of children could be evaluated in the MVPA category and 32% in physically inactive children according to WHO recommendations. More than 64% of all examined children were active before the pandemic from 3 to 36 months earlier (x = 15.41; SD = 10.29). In the parents’ opinion, children were active on average three times a week (1–7; SD = 1.83) regardless of gender (ANOVA, F = 1.61, *p* = 0.2). The average duration of PA ranged from 10 to 90 min (x = 38.44, SD = 23.43) and was directly proportional to age (*R* = 0.17, *p* < 0.0001). Most often it was spontaneous PA, such as outdoor activities, cycling, scooters, and ball games. Organized classes with a trainer systematically concerned dance (17.06%), soccer (15.16%), swimming (14.09%), acrobatic gymnastics (12.03%), and combat sports (4.65%). These classes lasted from 30 to 90 min, one to three times a week. During the pandemic, the PA of the children significantly decreased (*p* < 0.0001) (Figure 2).

**Figure 3:**
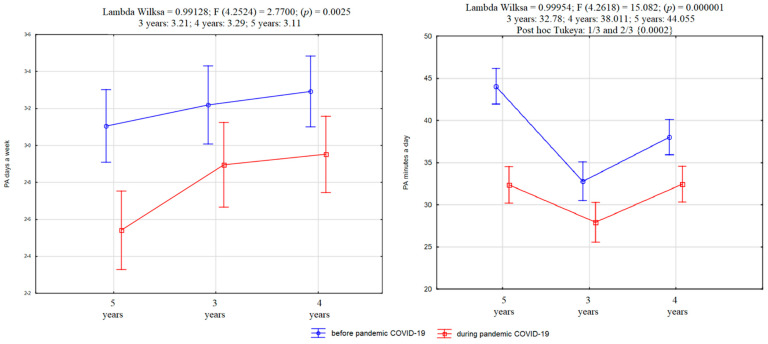
Average values of frequency (**left side**) and time of PA (**right side**) in groups of children aged three to five years.

More than 12% of children’s parents replaced their children’s motor activity with another form, realizing that it is essential in the process of motor development (3 yo: 9.75%; 4 yo: 11.58%; 5 yo: 15.16%; X^2^ = 5.48, df = 2, *p* = 0.06). Parents usually indicate cycling, scooters, jumping on a trampoline, family motor games, and dance parties. Time spent on these activities varies between 10 and 90 min (x = 30.99, SD = 23.6), which resulted in an average of 7.45 min shorter activity than before the pandemic (t = 12.96; *p* < 0.0001) (Figure 3).

### 3.4. Electronic Device Usage

**Figure 4:**
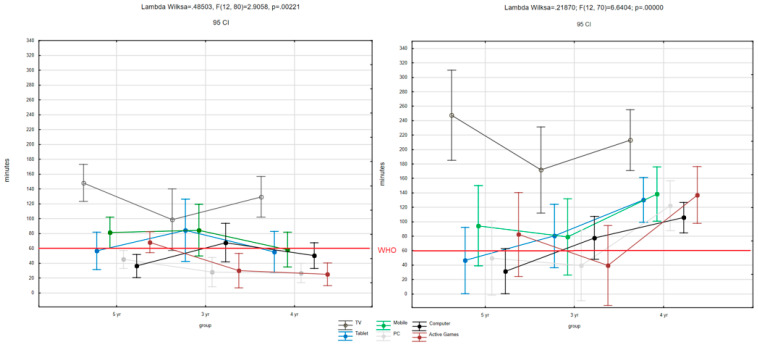
Average values of time spent using different electronic devices in groups of children aged 3–5 years (see before pandemic on the left side and during pandemic on the right side). Red line is the maximum time limit recommended by WHO for this age group.

Children used electronic devices for an excessive amount of time during the pandemic compared to before the pandemic (*p* < 0.00001). Analyzing all devices combined, 62.83% of three-year-olds, 66.67% of four-year-olds, and 85.49 of five-year-olds showed an increase in screen time. Stabilization was observed marginally in all groups (three yo: 11.79%; four yo: 14.01%; five yo: 3.08%), and decreases respectively at 25.38%, 19.32%, and 11.43%. Children are much more likely to use electronic devices in their free time. Children under five years of age used electronic devices on average 940.91 min/week before the pandemic vs. 1517.79 min/week during the pandemic. There was no difference, as expected, between boys and girls (*p* = 0.07), although differences were noted between age groups (Table 3). Among the devices most frequently used are TV, computer, parents’ smartphone, and a console or a tablet (Figure 4).

### 3.5. Sleeping Behavior

**Figure 5:**
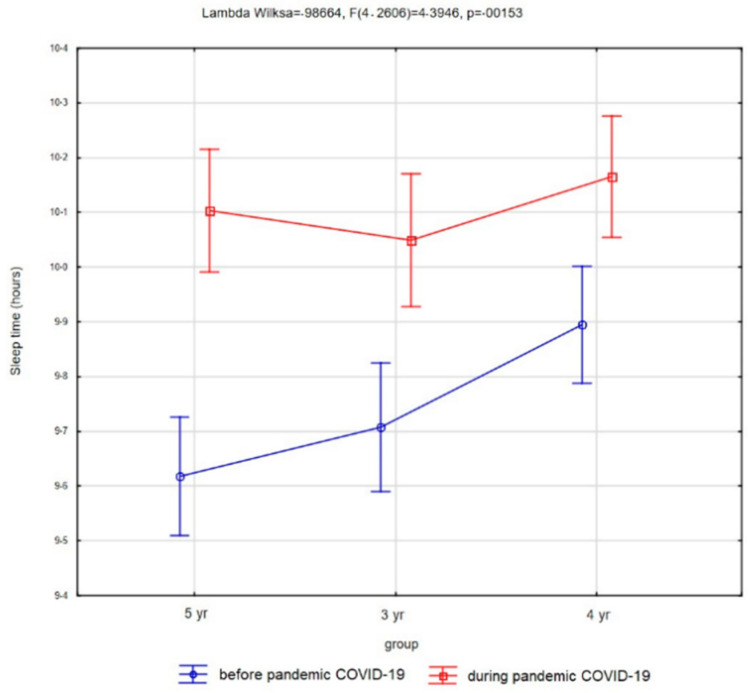
Average values of sleeping time in groups of children aged 3–5 years.

Children slept on average 9.74 h (SD, 1.18) before the pandemic and on average 10.11 h (SD, 1.21) during the pandemic (see Figure 5); the two measurements were statistically significantly different (t = 12.75, *p* < 0.00001). Before the pandemic 49.74% of three-year-olds slept with their parents in one bed, and this was 26.81% of four-year-olds, and 24.39% of five-year-olds. The pandemic period increased this habit by 11.28%, 6.59%, and 7.7%, respectively. During the pandemic period, 22.05% of three-year-olds, 22.34 four-year-olds, and 7.03% of five-year-olds had problems with falling asleep.

### 3.6. WHO Guidelines

It appeared that only 30.77% of children (24.62% of three-year-old, 38.97 % of four-year-old and 34.51% of five-year-old children) met the WHO guidelines before the pandemic complied with criteria. During the pandemic, the percentage of children meeting the recommendations of PA decreased. Children before the restrictions associated with the COVID-19 pandemic spent much more time in a sitting position than the one hour a day recommended by WHO. Before the pandemic, three-year-olds spent 4.36 h in the sitting position, four-year-olds spent 4.51 h and five-year-olds spent 4.91 h a day. During the pandemic, the time increased, and was respectively: 5.65, 6.15, and 6.59 h, respectively. The pandemic had a direct impact on the recommendations causing them to negatively increase or decrease regardless of the age of the child (*p* < 0.01). The vast majority of children slept 10–13 h a day as recommended, and the pandemic caused a positive percentage increase of 10–18%. Before the pandemic, most children, regardless of their age, had a limited time for electronic devices according to the WHO guidelines (1 h/day). Parents of three-year-olds declared 70.77% frequent or constant daily limits on the use of electronic devices, and this was 78.08% for 4-year-olds and 77.14% for 5-year-olds, respectively. These limits were mostly above WHO recommendations. These recommendations were only met by 35.48%, 26.38%, and 20.22% of three-, four-, and five-year-old children, respectively. During the pandemic, the children’s parents did not follow WHO recommendations on the use of electronic devices (*p* < 0.00001) (Table 4)

## 4. Discussion

In the research presented above, an attempt was made to evaluate the scale of the problem in Poland in terms of the variety of patterns and health behavior of children and possible changes in the time of limitations caused by the COVID-19 pandemic. In particular, the authors addressed the level of PA in children during the pandemic and as the volume of PA changed. Time spent sitting and the frequency of electronic device use and sleeping time were analyzed for the effect of COVID-19 virus pandemic-related restrictions on change. The benchmark for the analysis were the WHO guidelines for children under five years of age.

The level of PA has decreased. Our own studies but also studies by other authors clearly indicate a strong association between pandemic-related restrictions and reduced PA [1,2,4,12,13,14,15,17,18,20,23]. The COVID-19 lockdown has been seen to reduce the exercise time among children. Our studies have indicated that more than 30% of children could be evaluated in the MVPA category according to WHO recommendations and the same percentage of children were inactive. Whereas studies involving slightly older Canadian children (5–11 yo) conducted by Moore et. found that only 3.6% of children met the WHO recommended guideline for achieving 60 min of MVPA per day during the COVID-19 pandemic [12], compared with 12.7% meeting the guidelines reported by Rhodes et al. in 2019 [15]. This discrepancy is probably due to age differences between the study groups. The children in the study were younger and therefore the need for exercise for proper motor development was greater. It results from the predominance of the sympathetic over the parasympathetic nervous system [7].

Another Polish study on children and adolescents 6–17 years old found that the average value of the level of PA before the pandemic was four days with 60 min or longer PA, while during the pandemic it dropped to three days [29]. In our study the number of days spent on PA did not change significantly, but its time duration decreased to less than eight min on average.

Because many COVID-19 affected countries have closed schools, effective PA might not be sufficiently applied to children outside kindergartens [23,24]. It may be possible that children will be able to intuitively compensate for higher sedentary behaviors with more PA at home, but the current evidence remains inconclusive, and it may further depend on the setting characteristics [30,31]. It is clear from the research that, in lockdown, structured and controlled PA have been replaced by spontaneous PA, e.g., cycling, scooters, jumping on a trampoline, family motor games, and dance parties as in the study undertaken by Dunton et al. [32]. The time spent per week on outdoor PA was on average 56 min less per week, Okely et al. found [20] this time to be as much as 81 min less. It can be concluded that in both our own research and research conducted on children living in the U.S. [32], parents perceived children’s PA had decreased between the pre-COVID-19 period (February 2020) and during the pandemic (In U.S., April–May 2020 and in Poland May–June 2020).

Analyzing the results, it turned out that children spend much more time sitting during the COVID-19 pandemic compared to the pre-pandemic period and the positions taken are not always correct (slumped, asymmetrical positions). The problem really affects children all over the world, and our results are also consistent with a study conducted among children and adolescents in China [33] and in Germany [34]. Regardless of the period, about 50% of children were sitting in a cross-legged position (typical sit for preschool children, although debatable due to greatly changed lumbar and pelvic angles, and gluteal pressure [35], and the highest abduction angle from a seated position [36]). Our research shows that more than 60% of children’s parents pay attention to awkward sitting positions and the kids tend to get it from viewing friends or seeing it on the internet.

The sedentary behavior is associated with increased use of electronic devices [1,12,15,20]. The problem really affects children all over the world. Children and adolescents are increasingly using new information technologies to enrich their leisure time before the pandemic [10,22]. In Poland the amount of time spent on computer games also increased (1.29 vs. 1.64 h/day); however, using a smartphone on a regular day has decreased during the pandemic from 3.14 to 2.81 h per day [29]. Our own research shows that the children before the pandemic used the devices on average 2.24 h/day, increasing this time to 3.61 h/day. They are confirmed by the fears spread in many analyses of Western societies concerning parents’ lack of control over children’s time spent on using electronic devices. The leisure time was spent rather sedentary or with electronic devices, the use of which, without parental supervision, has increased drastically. Although, surprisingly, parents limit “definitely yes” the time spent using numerous and different electronic devices in more than 70% of each age group. The boys living in the U.S. spent more time than girls playing computer or video games, whereas girls spent more time than boys using the internet/emailing/electronic media for leisure [32]. In ow study was not fount this differences. The reason of this result is connected with the fact that sexual dimorphism is not marked until the school years [37].

Due to the fact that children are experiencing a change in their usual daily habits, it would be expected to find different sleep patterns; however, there is either moderate or insufficient evidence that daily habits such as higher screen time or fixed timetables substantially influence sleep [19]. Mori Stern et al. [38] and Paruthi et al. [39], point out the principles of sleep based on the American Academy of Sleep Medicine (AASM) recommendations According to these recommendations children three to five years of age should sleep 10 to 13 h per 24 h (including naps) on a regular basis to promote optimal health. Higher PA and outdoor play has been directly associated with longer sleep in preschoolers [19,30,39,40]. This assumption was not confirmed in a longitudinal study conducted by Okley et al. in 14 countries where it was found the children went to bed 34 min later and woke up 60 min later than before COVID-19. The mean nap time decreased by 19 min/day [20]. Dellaqiulia, reports that the amount of sleep time decreased and then stabilized during the pandemic [41]. In our study, different results were noted in children; the number of hours of sleep increased, but the sleep quality decreased. Children woke up more often during the night and therefore slept longer in the morning. It is difficult to discuss the reason for the different results due to the lack of detailed data on many variables, i.e., use of electronic devices just before bedtime, and the time of the last meal, which may influence the differences [19].

If we accept the above study results, we should ask a justified question: “Will the limitations of the coronavirus pandemic have a modifying effect on the wider sedentary lifestyle and what might be the consequences in the future in young children?” Dunton et al. point out that programmatic and policy strategies should be geared towards promoting PA and reducing sedentary behaviours over the next 12 months [32] and the results of their paper support such suppositions.

Given that the evidence on the effect of the COVID-19 pandemic outbreak on children’s PA level, screen time and sedentary habits of preschoolers is scarce and the study on the Polish population is the first in this area, this study has many of strengths. First, the sample was a nationally representative cohort of over 3000 parents of preschool children. Further, sampled parents represented the geographic, cultural, and socioeconomic makeup of Poland. Second, we assessed the status during the period of restrictions caused by the COVID-19 pandemic compared to the pre-pandemic period. Our study was limited by its questionnaire style, and as a result the possibility of recall bias may affect our findings. Although, given our aim, this approach was appropriate, especially since the research was conducted during the lockdown period, which was itself a huge limitation.

Results may not generalize to other countries. Increasing the availability of the analyzed data will be a necessary factor to maximize the value of these data and increase interest in the topic of sedentary lifestyles and behavior of children and the impact of the longer-term consequences of the COVID-19 pandemic on, for example, postural development and postural abnormalities. It is also crucial to develop recommendations for prevention management strategies and an optimal model of physioprophylaxis of sedentary lifestyles in the youngest group as an investment in a healthy society.

## 5. Conclusions

The results clearly indicate that the COVID-19 pandemic had an impact on changing patterns of behavior e.g., increased sedentary behaviour or decreased level of PA which resulted in increased time with electronic devices. During the pandemic, children had difficulty falling asleep and were more likely to sleep with their parents compared to the pre-restriction period. The percentage of children meeting WHO’s recommended guidelines decreased. Physical activity, sedentary time, and reduced PA will undoubtedly have long-term effects in the future. This project has a cognitive value in assessing the existing situation. The results could become the basis for developing recommendations for prevention management strategies of sedentary lifestyles in the youngest group.

## Figures and Tables

**Table 1:** Participants’ general characteristics and anthropometrics.

Parameters	Groups	Before Pandemic COVID-19 X (SD)	During Pandemic COVID-19 X (SD)	n ^I^	n ^II^	*p-*Values
Length (cm)	3 years{1}	109.56 (8.99)	109.64 (8.95)	388	388	0.00001 ^2^
	4 years {2}	109.91 (7.54)	109.95 (7.54)	470	468	0.000008 ^2^
	5 years {3}	118.68 (6.99)	118.75 (7.01)	455	455	0.00001 ^2^
Me (95%CI)		112 (112.36–113.32)	112 (112.42–113.38)			
*p-*Values		1/3; 2/3 {0.0000} ^1^	1/3; 2/3 {0.0000} ^1^			
Weight (kg)	3 years {1}	17.26 (3.63)	18.51 (3.74)	390	390	0.001 ^2^
	4 years {2}	17.51 (3.83)	18.77 (4.02)	471	471	0.001 ^2^
	5 years{3}	20.41 (3.72)	21.65 (3.94)	454	455	0.001 ^2^
Me (95%CI)		18 (18.22–19.60)	19 (19.46–19.91)			
*p-*Values		1/3; 2/3 {0.00002} ^1^	1/3; 2/3 {0.00002} ^1^			
BMI	3 years {1}	35.81 (34.8)	43.62 (35.02)	387	389	0.001 ^2^
	4 years {2}	30.61 (30.03)	40.48 (31.75)	471	470	0.001 ^2^
	5 years{3}	28.67 (26.53)	40.72 (32.46)	454	455	0.001 ^2^
Me (95%CI)		15 (29.76–33.2)	25 (39.72–43.28)			
*p-*Values		1/3 {0.005 } ^1^	0.32			
z-score	3 years {1}	25 (39.72–43.28)	−0.41 (1.66)	388	386	0.001 ^2^
	4 years {2}	−1.17 (1.47)	−0.43 (1.33)	471	469	0.001 ^2^
	5 years {3}	−1.13 (1.31)	−0.51 (1.28)	454	455	0.001 ^2^
Me (95%CI)		−1 (−1.22–−1.05)	0 (−0.53–−0.38)			
*p-*Values		0.74	0.48			

Data are mean (X) and standard deviations (SD); ^1^ Post-hoc by Tukey for ANOVA; ^2^ Wicoxona Test, z-scores for sexes combined, calculated by comparison with the WHO 2006 growth reference standards. Length refers to length-for-age; weight refers to weight-for-age. **Abbreviations:** {1}—three-years-old; {2}—four-years old; {3}—five-years-old; I—investigation before pandemic, II—investigation during pandemic; cm—centimeters; kg—kilograms; pr—percentiles; Me—Median; 95% CI—95% confidence intervals (CIs).

**Table 2:** Participants’ sitting time before and during the pandemic.

Sitting Time (Hours)	Groups	Before COVID-19 Pandemic X (SD)	During COVID-19 Pandemic X (SD)	n ^I^	n ^II^	*p-*Values
Sitting time (Monday–Friday)	3 years {1}	2.05 (1.07)	2.93 (1.54)	390	390	0.001 ^2^
	4 years {2}	2.02 (0.93)	3.11 (1.47)	471	470	0.001 ^2^
	5 years{3}	2.26 (1.04)	3.35 (1.43)	455	454	0.001 ^2^
Me (95%CI)		2 (2.06–2.17)	3 (3.05–3.22)			
*p-*Values		1/3 {0.01}; 2/3 {0.001} ^1^	1/3 {0.0002; 2/3 {0.04} ^1^			
Sitting time (Saturday–Sunday)	3 years {1}	2.32 (1.39)	2.75 (1.65)	389	390	0.00001 ^2^
	4 years {2}	3.12 (1.26)	2.48 (1.62)	471	470	0.001 ^2^
	5 years {3}	2.65 (1.31)	3.25 (1.50)	454	453	0.001 ^2^
Me (95% CI)		2 (2.42–2.54)	3 (2.94–3.11)			
*p-*Values		1/3 {0.001} ^1^	1/3 {0.00005; 2/3 {0.02} ^1^			

Data are mean (X) and standard deviations (SD); ^1^ Post-hoc by Tuckey for ANOVA; ^2^ Wicoxona Test. **Abbreviations:** {1}—three -years- old; {2}—four-years old; {3} —five-years-old; I—investigation before pandemic; II—investigation during pandemic; Me—Median; 95% CI—95% confidence intervals (CIs).

**Table 3:** Averages, standard deviations, and minimum and maximum values of days and time spent using electronic devices.

Type of Electronic Devices	Groups	Before COVID-19 Pandemic		n ^I^	During COVID-19 Pandemic		n ^II^	*p-*Values	
		Number of Days X ± SD (Range)	Minutes a Day X ± SD (Range)		Number of Days X ± SD (Range)	Minutes a Day X ± SD (Range)		Number of Days	Minutes a Day
TV	3 years {1}	5.17 ± 1.91 (1–7)	110.80 ± 54.03 (15–270)	365	5.55 ± 1.78 (1–7)	145.84 ± 91.19 (30–390)	379	0.00001 ^2^	0.00001 ^2^
	4 years {2}	5.37 ± 1.85 (1–7)	117.55 ± 52.99 (15–270)	458	5.86 ± 1.7 (1–7)	171.91 ± 82.18 (30–390)	461	0.00001 ^2^	0.01 ^2^
	5 years {3}	5.44 ± 1.85 (1–7)	121.21 ± 57.41 (15–270)	452	5.93 ± 1.61 (1–7)	162.76 ± 80.99 (30–390)	428	0.00001 ^2^	0.00001 ^2^
		*p* = 0.11	{1/3 = 0.03}^1^		{1/2 0.03} ^1^ {1/3 0.007} ^1^	{1/3 = 0.0003} ^1^ {1/2 = 0.00001} ^1^			
Tablet	3 years {1}	3.26 ± 1.81 (1–7)	72.38 ± 54.60 (15–240)	86	4.2 ± 1.83 (1–7)	99.12 ± 59.59 (15–240)	102	0.00001 ^2^	0.00005 ^2^
	4 years {2}	3.66 ± 2.01 (1–7)	63.81 ± 42.38 (15–240)	146	4.32 ± 2.15 (1–7)	94.76 ± 62.41 (15–240)	167	0.00001 ^2^	0.00001 ^2^
	5 years {3}	3.98 ± 2.05 (1–7)	76.38 ± 56.51 (15–270)	174	4.51 ± 2.14 (1–7)	101.49 ± 63.09 (15–240)	188	0.00001 ^2^	0.00001 ^3^
		{1/3 = 0.04} ^1^	*p* = 0.09		*p* = 0.41	p= 0.59			
PC	3 years {1}	3.64 ± 1.59 (1–7)	75.08 ± 65.02 (15–240)	44	3.27 ± 1.8 (1–7)	60.23 ± 32.13 (15–120)	64	0.06 ^3^	0.78 ^3^
	4 years {2}	2.54 ± 1.86 (1–7)	60.83 ± 55.37 (15–240)	90	3.18 ± 1.91 (1–7)	76.59 ± 69.47 (15–270)	122	0.0006 ^2^	0.43 ^2^
	5 years {3}	2.94 ± 2.08 (1–7)	63.16± 53.19 (15–240)	109	4.06 ± 2.14 (1–7)	72.08 ± 59.85 (15–240)	144	0.00001 ^2^	0.00002 ^2^
		{1/3 = 0.043 } ^1^ {1/2 = 0.01} ^1^	p= 0.37		{1/3 = 0.003} ^1^ {1/2 = 0.001} ^1^	*p* = 0.24			
Computer	3 years {1}	4.17 ± 2.29 (1–7)	43.59 ± 58.11 (10–180)	53	3.29 ± 1.93 (1–7)	92.5 ± 52.42 (15–180)	30	0.02 ^3^	0.26 ^3^
	4 years {2}	3.94 ± 2.19 (1–7)	30.64 ± 42.07 (10–180)	70	3.18 ± 1.58 (1–7)	76.25 ± 61.94 (15–180)	48	0.034 ^2^	0.35 ^3^
	5 years {3}	3.04 ± 1.54 (1–6)	27.29 ± 38.85 (10–210)	133	4.38 ± 2.07 (1–7)	72.43 ± 58.46 (15–240)	64	0.002 ^2^	0.00001 ^2^
		*p* = 0.04	*p* = 0.77		*p* = 0.86	*p* = 0.29			
Mobile	3 years {1}	3.72 ± 2.01 (1–7)	66.60 ± 42.48 (15–240)	184	4.47 ± 2.05 (1–7)	84.53 ± 65.86 (15–270)	192	0.00001 ^2^	0.00001 ^2^
	4 years {2}	3.75 ± 1.93 (1–7)	60.62 ± 40.74 (15–240)	221	4.13 ± 1.96 (1–7)	81.70 ± 70.27 (15–270)	238	0.01 ^2^	0.00002 ^2^
	5 years {3}	3.93 ± 2.18 (1–7)	68.96 ± 45.99 (15–240)	241	4.66 ± 2.17 (1–7)	86.01 ± 67.14 (15–270)	271	0.00001 ^2^	0.00001 ^2^
		*p* = 0.13	*p* = 0.11		{2/3 = 0.01}^2^	*p* = 0.59			
Active games	3 years {1}	3.38 ± 1.60 (1–6)	67.69 ± 37.21 (15–180)	39	3.49 ± 1.7 (1–7)	82.0 ± 70.03 (15–270)	45	0.006 ^3^	0.04 ^3^
	4 years {2}	3.53 ± 2.09 (1–7)	61.09 ± 40.53 (15–150)	69	3.72 ± 2.04 (1–7)	113.24 ± 90.63 (15–270)	71	0.00001 ^3^	0.01 ^3^
	5 years {3}	3.01 ± 1.89 (1–7)	77.5 ± 37.73 (15–150)	108	3.69 ± 2.06 (1–7)	99.78 ± 63.65 (15–270)	109	0.00001 ^2^	0.0002 ^2^
		*p* = 0.05	*p* = 0.02 ^1^ {2/3 = 0.03}		*p* = 0.76	*p* = 0.89			

Data are mean (X) and standard deviations (SD); range (minimum and maximum values); ^1^ Post-hoc by Tukey for ANOVA; ^2^ *p* for t-Student, **^3^** *p* for Wilcoxon test. **Abbreviations**: {1}—three -years- old; {2}—four-years old; {3}—five-years-old; I—investigation before pandemic; II—investigation during pandemic; TV—television; PC—Personal Computer.

**Table 4:** The numerical and percentage distribution of the examined children with and without WHO criteria in terms of PA, sitting, and sleeping hours.

WHO Guidelines	Groups	Before COVID-19 Pandemic		n ^I^	During COVID-19 Pandemic		n ^II^	Z
		YES * n (%)	NO * n (%)		YES * n (%)	NO *n (%)		
Physical Activity	3 years {1}	96 (24.61)	293 (75.13)	389	72 (18.46)	316 (81.03)	388	Z = 8.43; *p* = 0.00000 ^2^
	4 years {2}	152 (38.97)	318 (81.54)	470	104 (22.08)	365 (77.49)	469	
	5 years{3}	157 (34.51)	298 (65.49)	455	101 (22.20)	353 (77.58)	454	
ANOVA		F = 5.09; *p* = 0.006 ^1^ {1/2 = 0.008}			F = 1.13; *p* = 0.32			
Sitting Time	3 years {1}	90 (23.07)	300 (76.92)	390	56 (14.36)	333 (85.38)	389	Z = 11.61; *p* = 0.00000 ^2^
	4 years {2}	106 (22.51)	365 (77.49)	471	38 (8.07)	432 (91.72)	470	
	5 years{3}	92 (20.22)	363 (79.78)	455	35 (7.69)	420 (92.31)	455	
ANOVA		F = 0.58; *p* = 0.558			F = 6.61; *p* = 0.001 ^2^ {1/3 = 0.004}, {1/2 = 0.008}			
Sleeping Time	3 years {1}	226 (57.95)	160 (41.03)	389	276 (70.77)	114 (29.24)	389	Z = 10.06; *p* = 0.00000 ^2^
	4 years {2}	305 (64.76)	161 (34.18)	470	350 (74.31)	121 (25.69)	470	
	5 years{3}	269 (59.12)	186 (40.88)	455	356 (78.24)	99 (21.76)	455	
ANOVA		F = 2.76; *p* = 0.06			F = 3.12; *p* = 0.04 ^1^ {1/3 = 0.04}			
				
Electronic Devices Using	3 years {1}	138 (35.48)	251 (64.52)	389	66 (16.97)	323 (80.03)	389	
	4 years {2}	124 (26.38)	346 (73.62)	470	49 (10.43)	421 (89.57)	470	Z = 11.68; *p* = 0.00001 ^2^
	5 years{3}	92 (20.22)	363 (79.78)	455	29 (6.37)	426 (93.63)	455	
ANOVA		F = 12.69; *p* = 0.00001 ^1^ {1/3 = 0.000002}, {1/2 = 0.01}			F = 12.36; *p* = 0.00001 ^1^ {1/3 = 0.000002}, {1/2 = 0.009}			

Data given in numbers (*n*) and percentages (%); * YES = following the 2019 WHO recommendations, * NO = not in accordance with 2019 WHO recommendations; **^1^** Post-hoc by Tuckey for ANOVA; **^2^** for Wilcoxon Test. **Abbreviations:** {1}—three -years- old; {2}—four-years old; {3}—five-years-old; I—investigation before pandemic; II—investigation during pandemic.

## Data Availability

Data are archived at the Faculty of Health Sciences in Katowice, Medical University of Silesia in Katowice. If necessary, contact the first author by e-mail abrzek@sum.edu.pl.

## References

[1] Becker S.P., Gregory A.M. (2020). Editorial Perspective: Perils and promise for child and adolescent sleep and associated psychopathology during the COVID-19 pandemic. J. Child Psychol. Psychiatry Allied Discip..

[2] Lee J. (2020). Mental health E-ects of school closures during COVID-19. Lancet Child Adolesc. Health.

[3] Pietrobelli A., Pecoraro L., Ferruzzi A., Heo M., Faith M., Zoller T., Antoniazzi F., Piacentini G., Fearnbach S.N., Heymsfield S.B. (2020). Effects of COVID-19 Lockdown on Lifestyle Behaviors in Children with Obesity Living in Verona, Italy: A Longitudinal Study. Obesity.

[4] Bates L.C., Zieff G., Stanford K., Moore J.B., Kerr Z.Y., Hanson E.D., Barone Gibbs B., Kline C.E., Stoner L. (2020). COVID-19 Impact on Behaviors across the 24-Hour Day in Children and Adolescents: Physical Activity, Sedentary Behavior, and Sleep. Children.

[5] Willumsen J., Bull F. (2020). Development of WHO guidelines on physical activity, sedentary behavior, and sleep for children less than 5 years of age. J. Phys. Act. Health.

[6] WHO (2019). Guidelines on Physical Activity, Sedentary Behaviour and Sleep for Children Under 5 Years of Age: Web Annex: Evidence Profiles. https://apps.who.int/iris/handle/10665/311663.

[7] El-Sheikh M., Kouros C.D., Erath S., Cummings E.M., Keller P., Staton L. (2009). Marital conflict and children’s externalizing behavior: Interactions between parasympathetic and sympathetic nervous system activity. Monogr. Soc. Res. Child Dev..

[8] Stavridou A., Kapsali E., Panagouli E., Thirios A., Polychronis K., Bacopoulou F., Psaltopoulou T., Tsolia M., Sergentanis T.N., Tsitsika A. (2021). Obesity in children and adolescents during COVID-19 pandemic. Children.

[9] Choudhary M.S., Choudary A.B., Jamal S., Kumar R., Jamal S. (2020). the impact of ergonomics on children studying online during COVID-19 lockdown. J. Adv. Sports Phys. Educ..

[10] Brzęk A., Strauss M., Przybylek B., Dworrak T., Dworrak B., Leischik R. (2018). How does the activity level of the parents influence their children’s activity? The contemporary life in a world ruled by electronic devices. Arch. Med. Sci..

[11] World Health Organization (2017). Report of the Commission on Ending Childhood Obesity: Implementation plan: Executive Summary. https://apps.who.int/iris/handle/10665/259349.

[12] Moore S.A., Faulkner G., Rhodes R.E., Brussoni M., Chulak-Bozzer T., Ferguson L.J., Mitra R., O’Reilly N., Spence J.C., Vanderloo L.M. (2020). Impact of the COVID-19 virus outbreak on movement and play behaviours of Canadian children and youth: A national survey. Int. J. Behav. Nutr. Phys. Act..

[13] Guan H., Okely A.D., Aguilar-Farias N., del Pozo Cruz B., Draper C.E., El Hamdouchi A., Florindo A., Jauregui A., Katzmarzyk P., Kontsevaya A. (2020). Promoting healthy movement behaviours among children during the COVID-19 pandemic. Lancet Child Adolesc. Health.

[14] Fakhouri T.H.I., Hughes J.P., Brody D.J., Kit B.K., Ogden C.L. (2013). Physical activity and screen-time viewing among elementary school-aged children in the United States from 2009 to 2010. JAMA Pediatr..

[15] Rhodes R.E., Spence J.C., Berry T., Faulkner G., Latimer-Cheung A.E., O’Reilly N., Tremblay M.S., Vanderloo L. (2019). Parental support of the Canadian 24-h movement guidelines for children and youth: Prevalence and correlates. BMC Public Health.

[16] Is the Pandemic Having an Impact on the Way Children Sleep?. https://www.hopkinsallchildrens.org/ACH-News/General-News/Is-the-Pandemic-Having-an-Impact-on-the-Way-Childr%20%E2%80%8E.

[17] Aguilar-Farias N., Toledo-Vargas M., Miranda-Marquez S., Cortinez-O’Ryan A., Cristi-Montero C., Rodriguez-Rodriguez F., Martino-Fuentealba P., Okely A.D., del Pozo Cruz B. (2020). Sociodemographic Predictors of Changes in Physical Activity, Screen Time, and Sleep among Toddlers and Preschoolers in Chile during the COVID-19 Pandemic. Int. J. Env. Res. Public Health.

[18] Delisle Nyström C., Alexandrou C., Henström M., Nilsson E., Okely A.D., Wehbe El Masri S., Löf M. (2020). International Study of Movement Behaviors in the Early Years (SUNRISE): Results from SUNRISE Sweden’s Pilot and COVID-19 Study. Int. J. Env. Res. Public Health.

[19] Janssen X., Martin A., Hughes A.R., Hill C.M., Kotronoulas G., Hesketh K.R. (2020). Associations of screen time, sedentary time and physical activity with sleep in under 5s: A systematic review and meta-analysis. Sleep Med. Rev..

[20] Okely A.D., Kariippanon K.E., Guan H., Taylor E.K., Suesse T., Cross P.L., Chong K.H., Suherman A., Turab A., Staiano A.E. (2021). Global effect of COVID-19 pandemic on physical activity, sedentary behaviour and sleep among 3- to 5-year-old children: A longitudinal study of 14 countries. BMC Public Health.

[21] Zakrzewski-Fruer J.K., Gillison F.B., Katzmarzyk P.T., Mire E.F., Broyles S.T., Champagne C.M., Chaput J.P., Denstel K.D., Fogelholm M., Hu G. (2019). Association between breakfast frequency and physical activity and sedentary time: A cross-sectional study in children from 12 countries. BMC Public Health.

[22] King D.L., Delfabbro P.H., Billieux J., Potenza M.N. (2020). Problematic online gaming and the COVID-19 pandemic. J. Behav. Addict. J Behav. Addict..

[23] Tulchin-Francis K., Stevens W., Jr Gu X., Zhang T., Roberts H., Keller J., Dempsey D., Borchard J., Jeans K., VanPelt J. (2021). The impact of the coronavirus disease 2019 pandemic on physical activity in U.S. children. J. Sport Health Sci..

[24] Mazur J., Małkowska-Szkutnik A. (2018). Student Health in 2018 Against the New HBSC Screening Model.

[25] Marques A., Demetriou Y., Tesler R., Gouveia É.R., Peralta M., Matos M.G. (2019). Healthy lifestyle in children and adolescents and its association with subjective health complaints: Findings from 37 countries and regions from the HBSC Study. Int. J. Env. Res Public Health.

[26] TVN24. https://tvn24.pl/polska/koronawirus-w-polsce-szkoly-przedszkola-i-zlobki-zamkniete-w-calym-kraju-na-dwa-tygodnie-4344016?fbclid=IwAR3fNXmzl63tIFE9Ie6UTTO6h4QdZx9WQXws7b0Dq5Lpi2ROr4rTttYan3w.

[27] de Onis M., Onyango A.W., Borghi E., Siyam A., Nishida C., Siekmann J. (2007). Development of a WHO growth reference for school-aged children and adolescents. Bull. World Health Organ..

[28] Cole T.J., Lobstein T. (2012). Extended international (IOTF) body mass index cut-offs for thinness, overweight and obesity. Pediatr. Obes..

[29] Łuszczki E., Bartosiewicz A., Pezdan-Śliż I., Kuchciak M., Jagielski P., Oleksy Ł., Stolarczyk A., Dereń K. (2021). Children’s Eating Habits, Physical Activity, Sleep, and Media Usage before and during COVID-19 Pandemic in Poland. Nutrients.

[30] López-Bueno R., López-Sánchez G.F., Casajús J.A., Calatayud J., Tully M.A., Smith L. (2021). Potential health-related behaviors for pre-school and school-aged children during COVID-19 lockdown: A narrative review. Prev. Med..

[31] Grao-Cruces A., Segura-Jiménez V., Conde-Caveda J., García-Cervantes L., Martínez-Gomez D., Keating X.D., Castro-Piñero J. (2019). The role of school in helping children and adolescents reach the physical activity recommendations: The UP&DOWN Study. J. Sch. Health.

[32] Dunton G.F., Do B., Wang S.D. (2020). Early effects of the COVID-19 pandemic on physical activity and sedentary behavior in children living in the U.S.. BMC Public Health.

[33] Xiang M., Zhang Z., Kuwahara K. (2020). Impact of COVID-19 pandemic on children and adolescents’ lifestyle behavior larger than expected. Prog. Cardiovasc. Dis..

[34] Schmidt S.C.E., Anedda B., Burchartz A., Eichsteller A., Kolb S., Nigg C., Niessner C., Oriwol D., Worth A., Woll A. (2020). Physical activity and screen time of children and adolescents before and during the COVID-19 lockdown in Germany: A natural experiment. Sci. Rep..

[35] Yu J.S., An D.H. (2015). Differences in lumbar and pelvic angles and gluteal pressure in different sitting postures. J. Phys. Ther. Sci..

[36] Ganokroj P., Chaowalitwong J., Kerdsomnuek P., Sudjai N., Lertwanich P., Vanadurongwan B. (2021). Three-dimensional motion analysis of ten common Asian sitting positions in daily living and factors affect range of hip motions. BMC Musculoskelet. Disord..

[37] Tatarczuk J., Choptiany M. (2019). Sexual dimorphism in selected morphological features. Physical Activity of People of Different Ages.

[38] Stern M., Wagner M.H., Thompson L.A. (2020). Current and COVID-19 challenges with childhood and adolescent sleep. JAMA Pediatr..

[39] Paruthi S., Brooks L.J., D’Ambrosio C., Hall W.A., Kotagal S., Lloyd R.M., Malow B.A., Maski K., Nichols C., Quan S.F. (2016). Recommended amount of sleep for pediatric populations: A consensus statement of the American Academy of Sleep Medicine. J. Clin. Sleep Med..

[40] Belmon L.S., van Stralen M.M., Busch V., Hamsen I.A., Chinapaw M.J.M. (2019). What are the determinants of children’s sleep behavior? A systematic review of longitudinal studies. Sleep Med. Rev..

[41] Dellagiulia A., Lionetti F., Fasolo M., Verderame C., Sperati A., Alessandri G. (2020). Early impact of COVID-19 lockdown on children’s sleep: A 4-week longitudinal study. J. Clin. Sleep Med..

